# Long-term follow-up of children with chronic non-bacterial osteomyelitis—assessment of disease activity, risk factors, and outcome

**DOI:** 10.1186/s13075-023-03195-4

**Published:** 2023-11-28

**Authors:** Christiane Reiser, Jens Klotsche, Toni Hospach, Georg Heubner, Daniel Windschall, Ralf Trauzeddel, Nadine Groesch, Martina Niewerth, Kirsten Minden, Hermann Girschick

**Affiliations:** 1 Department of Pediatrics, Landeskrankenhaus Bregenz, Bregenz, Austria; 2 Department of Pediatrics, Division of Pediatric Rheumatology and autoinflammation reference center Tuebingen (arcT), Tuebingen, Germany; 3 Deutsches Rheuma-Forschungszentrum Berlin, ein Institut der Leibniz-Gemeinschaft, Berlin, Germany; 4 Department of Pediatrics, Olgahospital, Klinikum Stuttgart, Stuttgart, Germany; 5 Städtisches Klinikum Dresden-Neustadt, Klinik für Kinder- und Jugendmedizin, Dresden, Germany; 6 Clinic for Pediatric and Adolescent Rheumatology, St. Josef-Stift, Sendenhorst, Germany; 7 University of Halle –Wittenberg, Halle, Germany; 8 Fachambulanz Kinderrheumatologie, Helios Klinikum Berlin-Buch, Klinik für Kinder- und Jugendmedizin, Berlin, Germany; 9 Department of Pediatric Respiratory Medicine, Immunology and Critical Care Medicine, Charité – Universitätsmedizin Berlin, corporate member of Freie Universität Berlin and Humboldt Universität zu Berlin, Berlin, Germany; 10 Vivantes Klinikum Friedrichshain, Children’s Hospital, Berlin, Germany; 11 German Center for Growth and Development DeuzWeg, Berlin, Germany; 12 Childrens’ Hospital, University of Wuerzburg, Wuerzburg, Germany

**Keywords:** Chronic nonbacterial osteomyelitis, Chronic recurrent multifocal osteomyelitis, Long-term follow-up, Longitudinal registry, Remission, Disease activity score, PedCNO score

## Abstract

**Introduction:**

Chronic non-bacterial osteomyelitis (CNO) is an autoinflammatory bone-disease of unknown origin. The National Pediatric Rheumatologic Database (NPRD) collects long-term data of children and adolescents with rheumatic diseases including CNO.

**Objective:**

To assess characteristics, courses, and outcomes of CNO with onset in childhood and adolescence and to identify outcome predictors.

**Methods:**

From 2015 to 2021 patients with a confirmed diagnosis of CNO, who were registered in the NPRD during their first year of disease and at least one follow-up visit, were included in this analysis and observed for up to 4 years.

**Results:**

Four hundred patients with recent diagnosis of CNO were enrolled in the NRPD during the study period. After 4 years, patient data documentation was sufficient to be analyzed in 81 patients. A significant decline of clinical and radiological lesions is reported: at inclusion in the registry, the mean number of clinical lesions was 2.0 and 3.0 MRI lesions per patient. A significant decrease of manifestations during 4 years of follow-up (mean clinical lesions 0.5, *p* < 0.001; mean MRI lesions 0.9 (*p* < 0.001)) was documented. A significant improvement of physician global disease activity (PGDA), patient-reported overall well-being, and childhood health assessment questionnaire (C-HAQ) was documented. Therapeutically, an increase of disease-modifying anti-rheumatic drugs over the years can be stated, while bisphosphonates rather seem to be considered as a therapeutic DMARD option in the first years of disease. Only 5–7% of the patients had a severe disease course as defined by a PGDA >  = 4. Predictors associated with a severe disease course include the site of inflammation (pelvis, lower extremity, clavicle), increased erythrocyte sedimentation rate, and multifocal disease at first documentation. The previously published composite PedCNO disease activity score was analyzed revealing a PedCNO70 in 55% of the patients at 4YFU.

**Conclusion:**

An improvement of physician global disease activity (PGDA), patient reported overall well-being and imaging-defined disease activity measures was documented, suggesting that inactivity of CNO disease can be reached. PedCNO score and especially PGDA, MRI-defined lesions and in a number of patients also the C-HAQ seem to be reliable parameters for describing disease activity. The identification of risk factors at the beginning of the disease might influence treatment decision in the future.

**Supplementary Information:**

The online version contains supplementary material available at 10.1186/s13075-023-03195-4.

## Background/introduction

CNO is an autoinflammatory bone disease, primarily affecting children. It was first described by Gideon et al. [1]. Recently, first-year disease data from patients with chronic non-bacterial osteomyelitis (CNO) enrolled in the National Paediatric Rheumatology Database (NPRD) over a 10-year period were presented [2]. Sociodemographic and clinical parameters of pediatric rheumatologic diseases are documented in the NPRD on a yearly basis via questionnaires. These are answered by referral center pediatric rheumatologists and patients/parents. For details of NPRD set up, inclusion of patients, and yearly documentation, we refer to [2, 3]: in the first year of documentation, a favorable therapeutic response was documented based predominantly on the usage of non-steroidal anti-inflammatory drugs (NSAID). Over the inclusion period of 10 years (2009–2018), changes in the diagnostic and therapeutic strategy could be noted, e.g., imaging lately relies primarily on whole-body MRI imaging. Even though CNO still is a diagnosis of exclusion and no validated classification exists, physicians’ awareness and confidence in making the diagnosis based on the clinical features and imaging has improved significantly. In pediatric rheumatology, CNO is regularly seen and not as rare as formerly supposed [4, 5]. Thus, the numbers of biopsies reported in NPRD has dwindled [2]. Compared to previous national and international cohorts, the mean number of clinical and radiological bone lesions was lower in patients recorded in the NPRD database [4–9]. From previous cohorts [2, 10], it was well known that especially in the early few months of NSAID treatment, a significant effect of improvement can be noted. Especially, the clinical parameters patients’ overall well-being and pain as well as—to some extent—imaging (MRI) lesions may improve rapidly. Since controlled studies on treatment are lacking and no approved medication for CNO exists, treatment modalities and outcome after treatment can only be estimated from observational studies and disease registers, currently. Within the first 12 months, a significant improvement was documented clinically in about one third of the patients; complete MRI resolution as defined by zero lesions is found in 17% of the patients [2, 10]. Whether patients’ remission further improves over time is of particular interest for the long-term analysis. These findings will be compared to previous retrospective international cohorts. The French cohort documented that two thirds of patients still have active disease after 4 years [7].

The objective of the current analysis was to show demographic, clinical, imaging data, disease course after treatment initiation, and outcome predictors.

## Patients and methods

### Registry and questionnaires

The NPRD generally collects data from pediatric patients with inflammatory diseases being followed in pediatric rheumatology centers. Once a year, patients/parents and their physicians answer a standardized questionnaire parallelly [2]. More than 60 pediatric rheumatology centers in Germany and Austria participated in the NPRD and recorded patients with CNO once a year for this study (see list in the Additional file 5). Patients were selected and submitted into the database by on site expert-confirmed diagnosis of non-bacterial osteomyelitis based on clinically symptomatic inflammatory bone lesions after exclusion of bacterial, syndromic, or oncological differential diagnoses. Data of individual patients submitted to the database was reviewed for plausibility and exclusion criteria and subjected to long-term analysis by CR, KM, and HG. Patients enrolled in the NPRD between 2015 and 2020 who had a disease duration of ≤ 12 months and who had at least one follow-up during the following 4 years were included in the long-term analysis [2]. One target of the CNO registry is to expand the knowledge on long-term follow-up CNO data. Findings might assist in treatment decisions and counseling of patients and their families.

In this study, two standardized questionnaires were used (details please refer to [2]): the patient’s questionnaire includes the German version of the Childhood Health-Assessment Questionnaire (C-HAQ), patient-reported overall well-being, and pain, each on a 21-point numerical rating scale from 0 to 10 (NRS) [11]. The doctor’s questionnaire includes a variety of clinical and sociodemographic features like physician´s global assessment of disease activity (PGDA, NRS), clinical number of bone lesions, number of lesions defined by MRI, and laboratory parameters such as ESR (erythrocyte sedimentation rate) as well as treatment modalities. The numbers of clinical co-manifestations like arthritis, sacroiliitis as defined clinically and by whole-body MRI (WB-MRI), inflammatory skin disease (psoriasis, acne, palmoplantar pustulosis), or chronic inflammatory bowel disease were reported. The fulfillment of classification criteria of enthesitis-related arthritis or psoriatic arthritis based on ILAR criteria was questioned [12].

### Definitions of outcome

Disease activity was categorized as follows: inactive disease: PGDA < 1; mild disease: PGDA 1–3; severe disease: PGDA ≥ 4. The composite response PedCNO score [10] was calculated from baseline to each follow-up assessment. It was used to calculate disease activity and treatment response over time. The PedCNO score consists of 5 variables: ESR, number of MRI defined radiological lesions, physician global assessment (PGDA; NRS), patient global assessment on overall well-being (NRS), and C-HAQ. Out of these 5 variables, score categories of 30%, 50%, and 70% improvement of variables were calculated [8].

### Statistical analysis

Standard descriptive statistics were used to describe the distribution of sociodemographic, clinical, and imaging parameters. Pre-analyses of data were performed to investigate a possible attrition bias due to loss of follow-up. We could not detect a statistically significant association between collected parameters such as variables covering disease severity at baseline and the likelihood of loss of follow-up. Age- and gender-specific percentiles were calculated based on those of a German reference population [13]. Longitudinal data were analyzed by generalized linear mixed models including time and number of lesions at the onset of disease and HLA-B27 positivity besides other variables of interest as covariates. Linear mixed models were used for continuously distributed response variables (e.g., physician’s global assessment of disease activity on NRS) and logistic mixed model for binary response variables (e.g., number of patients with no functional limitations by C-HAQ). Statistical analyses were performed with SAS 9.3.

## Results

### Patient characteristics

Patients documented from 2015 to 2021 (1618 patients, 3168 visits) were the base for this analysis. Among those, 400 patients were eligible for the current longitudinal analysis. Patient characteristics are given in Table 1.

**Table 1:** Baseline and follow-up features (4 years) of the patients in the registry

	At baseline	4-year follow-up
Total no. of eligible patients with follow-up data available	400	81
Female, *n* (%)	258 (65.5%)	47 (58.0%)
Age at disease onset in years, mean (SD)	11 years (SD 2.9)	-
Time between symptom onset and first visit to pediatric rheumatology in months, mean	5.3 months (SD 5.2)	-
Inclusion to the registry after first visit to pediatric rheumatology, mean	5.8 months (SD 3.2)	-
HLA-B27 positive^a^	21 (13.2%)	5 (13.5%)
Physician’s global assessment, NRS, mean (SD)^b^	2.0 (1.9)	0.9 (1.7)
Patient’s global assessment, NRS, mean (SD)^b^	2.5 (2.4)	2.2 (2.5)
Patients’ pain, NRS, mean (SD)^b^	2.6 (2.7)	1.9 (2.6)
C-HAQ, NRS, mean (SD)^b^	0.3 (0.4)	0.2 (0.3)
Arthritis	79 (20.8%)	13 (16.9%)
-Peripheral	64 (16.9%)	9 (11.7%)
-Sacroiliitis	20 (5.3%)	4 (5.2%)
Number of patients (%) meeting the classification criteria for		
-Psoriatic arthritis	5 (1.3%)	3 (4.0%)
-Enthesitis-related arthritis	3 (0.8%)	3 (4.0%)
Skin disease (psoriasis, acne, PPP)	61 (15.6%)	14 (17.7%)
IBD	2 (0.5%)	5 (6.6%)

*PPP* pustulosis palmoplantaris, *IBD* inflammatory bowel disease, *No.* number

^a^HLA-B27 determined in 159 patients at baseline and in 37 at 4-year follow-up

^b^At inclusion into the registry (baseline)

After 4 years of follow-up, 20% of the patients were still documented in the registry, 28% of them with active disease as defined by a disease activity ≥ 1 via NRS by the physician. Risk for concomitant arthritis was higher in patients with an initial “higher” number of bone-lesions (OR 1.18, *p* = 0.016).

When considering different skin manifestations like *psoriasis* and *acne*, no significant change in frequency was noted. Their proportion ranged between 15.6% (baseline) and 19.4% (1YFU) to 17.7% (4YFU) of patients. Of note, pustulosis palmoplantaris significantly increased from initially 4.6% of patients to 7.8% after 1 year and to 8.7% after 3 years (*p* = 0.041) (Table 1). The proportion of patients with *hyperostosis at any site* did not change over time, ranging from 17.3 to 12.4% of patients. In addition, those with *spinal fractures/compressions* during follow-up did not change (4.5%).

### Anthropomorphic data comparison to the national reference cohort during follow-up

**Fig. 1:**
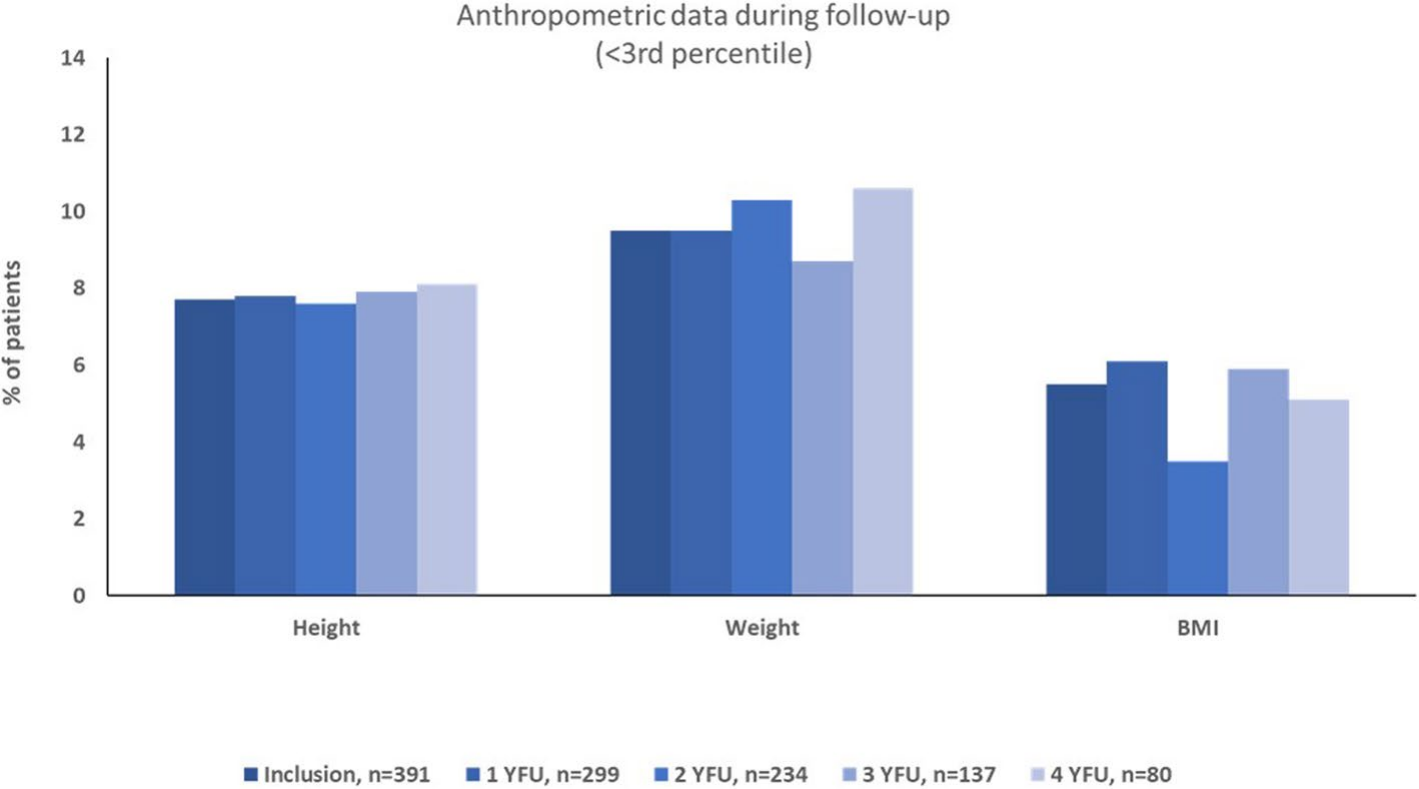
Percentage of patients with length, weight, and body mass index below the 3rd percentile at inclusion and during follow-up. BMI, body mass index; YFU, year follow-up. Inclusion is in average 5.8 months after first visit to pediatric rheumatology

At 4-year follow-up, still 8% of patients exhibited a height below 2 standard deviations, 11% a weight below 2 standard deviations. Consistently, 5% of patients had a BMI below the 3rd percentile over time. There was no significant change in the proportion of patients with height, weight, or BMI below 2 standard deviations over time (Fig. 1). No relevant co-diagnoses like celiac disease were documented.

### Distribution of osteomyelitic lesions during disease course

At initial documentation, the majority of patients had clinically defined lesions located in the tibia, femur, pelvis, clavicle, and the vertebral bodies (32%; 28%; 23%; 21%; 15% respectively). During follow-up, these lesions stayed predominant. Only 21 patients had *clinically* active lesions after 4 years, which were located mainly on tibia and pelvis (each 29%), femur, and clavicle (each 19%). Considering the distribution of the bone manifestations on a basis of *all lesions* after 4 years, 18% of all lesions were in the tibia, 13% in femur, 15% each in pelvis and clavicle, and 5% in the vertebral body. Of interest, hardly any lesion was detected in the upper extremity, the ribs, and the patella after 2 years. Over time manifestations focused on the lower extremity (femur, tibia, fibula, metatarsalia, calcaneus), pelvis, and clavicle.

**Fig. 2:**
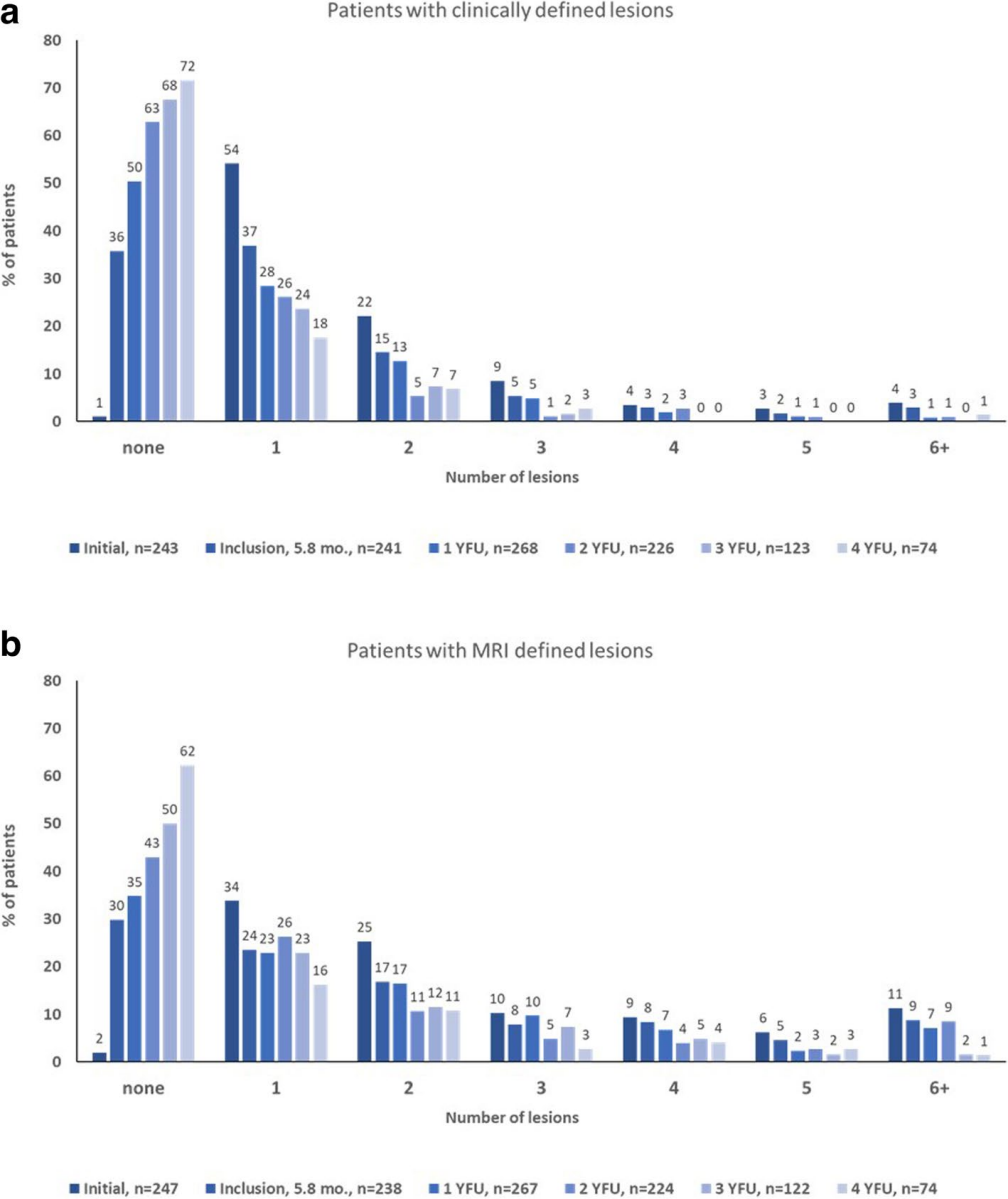
Number of active bone lesions over time. **a** Patients with number of clinical lesions during disease course. **b** Patients with MRI defined lesions. YFU, year follow-up

Considering the reduction of lesions as defined by imaging, 21% of patients showed vertebral manifestations at baseline. This proportion stepwise decreased over time, ending up with 2.7% of all patients after 4 years. Depicted in Fig. 2, a steady decline during continuous therapy/follow-up of clinically overt lesions was noted. This reduction could be seen in any patients regardless of initial number of lesions (Fig. 2a, b). MRI imaging technique identified higher numbers of lesions during follow-up compared to clinical notification (Fig. 2b). After 4 years, more than two thirds of patients (71.6%) were clinically symptom-free. 62.2% of patients were without MRI manifestations. Even after several years of follow-up, a further improvement over time was still measurable.

**Fig. 3:**
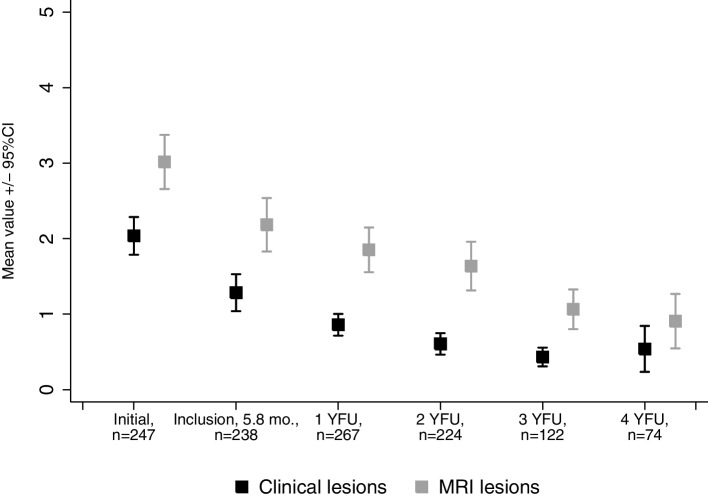
Mean number of clinically overt and MRI lesions per patient. YFU, year follow-up. Data shown by means with 95% confidence interval of the mean

By analyzing the mean of lesions from a combined clinical and imaging perspective, the mean number of lesions was 2.0 (standard deviation (sd) 2.0) clinical lesions and 3.0 (sd 2.9) MRI-defined lesions per patient at disease onset, with significant decrease of manifestation sites until 4-year follow-up (mean clinical lesions 0.5 (1.3), *p* < 0.001; mean lesions defined by imaging 0.9 (1.6, *p* < 0.001) ((Fig. 2a + b) and Fig. 3).

### Long-term therapeutic strategies

Most patients (*n* = 387/400; 97%) received medication at the time of first documentation/inclusion into the registry. The proportion of patients *off* medication increased significantly over time (3.2% at inclusion to 27.2% (after 4-year follow-up) (*p* < 0.001)). Bisphosphonate usage seemed to be predominantly instituted at the beginning of CNO therapy; the reported usage steadily declined from 9 to 4% of patients. Bisphosphonates are usually administered intravenously. Two regimes are generally used in Germany: regime 1 with three consecutive days of, e.g., pamidronate once a day vs. regime 2 with one infusion of bisphosphonates once a month. The duration of therapy is mostly 6–9 months [14, 15]. Of those receiving medication after 4 years (*n* = 59 patients on medication), roughly 54% were on NSAIDs and 36% were on b- or csDMARDs (methotrexate most common) (see Table 2). Further details are listed in Table 2. Treatment regimes of DMARDs were according to JIA standard protocols.

**Table 2:** Therapies applied at inclusion in the registry and at 4-year follow-up

	At baseline *N* = 400	After 4 years *N* = 81 (all patients)	
Any medication	387 (96.8%)	59 (72.8%)	*p* < 0.001
NSAIDs	344 (86%)	32 (39.5%)	*p* < 0.001
Systemic glucocorticoids, total	46 (11.5%)	3 (3.7%)	*p* = 0.003
< 0.2 mg per kg BW	21 (5.3)	2 (2.5%)	*p* = 0.285
≥ 0.2 mg per kg BW	33 (8.3%)	2 (2.5%)	*p* = 0.068
Bisphosphonates	34 (8.5%)	3 (3.7%)	*p* = 0.140
DMARDs, total^a^	56 (16.4%)	21 (28.4%)	*p* = 0.008
csDMARDs	43 (12.6%)	13 (17.6%)	*p* = 0.129
Methotrexate	30 (8.8%)	10 (13.5%)	*p* = 0.21
Sulfasalazine	13 (3.8%)	3 (4.1%)	*p* = 0.836
bDMARD	21 (6.1%)	9 (12.2%)	*p* = 0.003
Etanercept	10 (2.9%)	3 (4.1%)	*p* = 0.542
Adalimumab	11 (3.2%)	6 (8.1%)	*p* = 0.05

*NSAIDs* non-steroidal anti-rheumatic drugs, *csDMARDs* conventional synthetic disease-modifying anti-rheumatic drugs, *bDMARDs* biological DMARDs, *BW* body weight

^a^Information on DMARD therapy was available for 341 patients at baseline and 74 patients at 4-year follow-up, respectively

### Changes in physician- and patient-reported outcomes

**Fig. 4:**
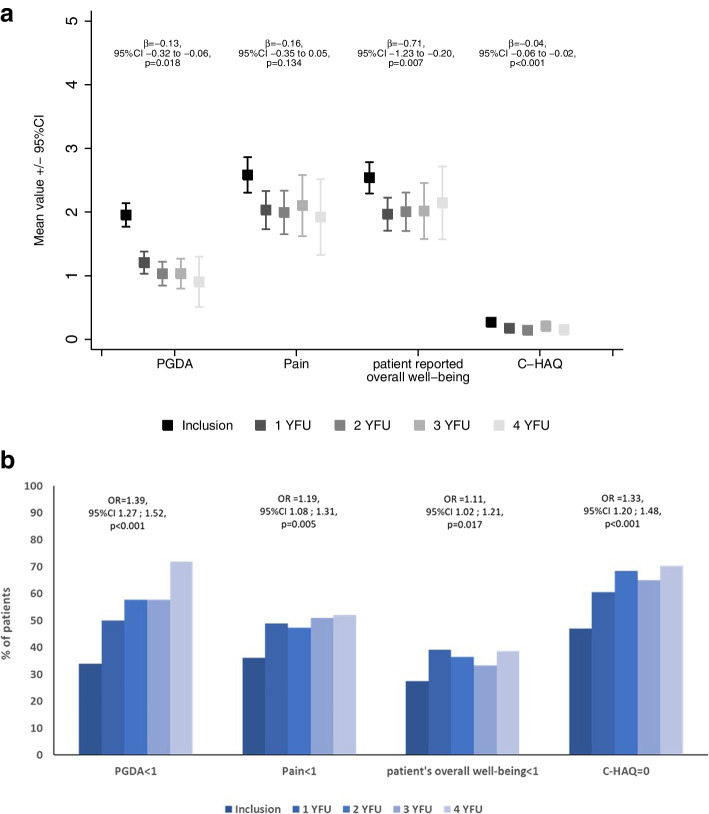
**a** Physician- and patient-reported outcomes over time: NRS, numeric rating scale; C-HAQ, childhood- health assessment questionnaire; PGDA, physician global disease activity. Data shown by means with 95% confidence interval of the mean (95% CI C-HAQ: inclusion 0.23–0.31; 1 YFU 0.13–0.21; 2 YFU 0.10–0.19; 3 YFU 0.13–0.28; 4 YFU 0.07–0.23). **b** Number of patients with favorable outcomes. Physician global disease activity (PGDA) NRS < 1, patient pain NRS < 1, patient overall well-being NRS < 1, and C-HAQ = 0 (childhood health assessment questionnaire)) from inclusion to 4 years of follow-up; percentages of patients are given, who reached the proposed levels of remission. OR, odds ratio

We found a *significant improvement* over time in *PGDA* (*p* = 0.018), *patient overall well-being* (*p* = 0.007), and *C-HAQ* (*p* < 0.001) considering the outcome parameters PGDA, patient-reported pain, patient-reported overall well-being, and physical function (C-HAQ score) throughout follow-up. The change in patient-reported pain also decreased without reaching statistical significance (*p* = 0.134) (Fig. 4).

Regarding clinical parameters, a significant increase in patients with inactive disease, without pain, and unimpaired well-being was seen (Additional file 1). When a PGDA < 1 was considered, the increase of clinically inactive disease patients was significant (*p* < 0.001). In fact, the number of patients with inactive disease raised from 34 to 72% at 4-YFU (Additional file 1). Also, a C-HAQ level of zero was repeatedly demonstrated in an increasing number of patients over time (*p* < 0.001). It is of relevance that 47% of patients already reported a C-HAQ level of zero at inclusion/baseline (Additional file 2). However, for the remaining 53% of patients, the C-HAQ seems to be a responding score to describe disease activity over time. A C-HAQ score of zero during follow-up was unlikelier when higher numbers of lesions were detected at baseline (OR 1.33, 95% CI 1.20; 1.48, *p* < 0.001).

### Correlations for severe disease course

The definition of potential correlates for severe disease course is needed to identify patients, who might need early escalation of therapy in the future. A total of 8.6%, 6.8%, 6.7%, and 5.3% of the patients were considered by their physicians to have “severe disease” (PGDA ≥ 4) at 1-, 2-, 3-, and 4- year follow-up (Table 3). When this fraction was compared from baseline to the 4 YFU, a significance decrease was found (*p* = 0.000017). The following clinical, imaging, and laboratory parameters at first documentation were associated with a PGDA ≥ 4 over the complete follow-up time.

**Table 3:** Development of disease activity categories scored by rheumatologist PDGA

	Baseline		1st follow-up		2nd follow-up		3rd follow-up		4th follow-up	
	*N*	%	*N*	%	*N*	%	*N*	%	*N*	%
Inactive disease (NRS < 1)	130	33.9	148	50.0	135	57.7	78	57.8	54	72.0
Mild disease activity (NRS 1–3)	183	47.7	122	41.2	83	35.5	48	35.6	17	22.7
Severe disease activity (NRS ≥ 4)	71	18.5	26	8.8	16	6.8	9	6.7	4	5.3

Severe disease activity compared from baseline to the 4 YFU: *p* = 0.000017

*NRS* numerical rating scale

It was calculated that an ESR increase by 1 mm/h at inclusion may rise the risk for severe disease by 3%. For each additional MRI lesion, the risk for severe disease (PGDA ≥ 4 over time) was elevated by 19%. If the pelvis, femur, or clavicle were affected at baseline, the likelihood of PGDA ≥ 4 was significantly increased (55%, 47%, 68%, respectively). The highest risk elevation for severe disease course of almost 150% was observed in multifocal (defined as lesions ≥ 2) CNO disease onset (CRMO disease course) (Table 4). No significant association for *severe disease activity* was present for gender, height below the 3rd percentile, or lesions in the calcaneus or tibia. The presence of HLA-B27 had been associated with a higher number of lesions in the 1st year analysis without reaching significance [2]. In the long-term analysis, however, its presence was *not* significantly associated with a severe course of disease.

**Table 4:** Predictors for severe disease course as indicated by PGDA ≥ 4

	OR^2^	95% CI	*p* value
Female	1.24	0.78; 1.97	0.371
ESR; one additional mm/h	**1.03**	**1.01; 1.05**	**0.006**
CRP, one additional mg/l	1.05	0.99; 1.09	0.068
One additional radiological lesion	**1.19**	**1.08; 1.31**	** < 0.001**
Clavicle	**1.68**	**1.12; 2.53**	**0.012**
Pelvis	**1.55**	**1.21; 1.97**	**0.005**
Femur	**1.47**	**1.15; 1.89**	**0.002**
Tibia	1.21	0.91; 1.61	0.184
Calcaneus	1.38	0.89; 2.15	0.151
Multifocal	**2.48**	**1.41; 4.38**	**0.002**

OR was estimated by generalized estimation equations

*OR* odds ratio, *CI* confidence interval, *ESR* erythrocyte sedimentation rate

### PedCNO score

Table 5 reports that already after 1-year, half of patients improved by 30%, 47% by 50%, and 41% of patients by 70% of these core variables. Over the years, further improvement was noted, resulting in PedCNO70 in 55% of patients at 4 YFU (Table 5).

**Table 5:** PedCNO score in the current analysis

PedCNO-score category	30	50	70
Beck 2010 [10]	62%	57%	54%
Current analysis:			
1-year FU (*n* = 305)	149 (49%)	143 (47%)	125 (41%)
2-year FU (*n* = 240)	144 (60%)	132 (55%)	110 (46%)
3-year FU (*n* = 137)	79 (58%)	66 (48%)	59 (43%)
4-year FU (*n* = 81)	58 (72%)	54 (67%)	45 (55%)

*FU* follow-up

## Discussion

In the current prospective long-term follow-up analysis, we described the outcome of 400 CNO patients up to 4 years, disease trajectories, and factors associated with the outcome.

We compared the NPRD to other published CNO cohorts [2, 4, 6–9, 16]. We found a slightly lower proportion of patients with multifocal disease as a potential marker of disease severity (clinically 48% of patients and MRI defined 65% of patients compared to 57–93% [7, 16] in other cohorts (Additional file 3)). Patients with active disease at final follow-up range from 22 to 66% in the Huber and Wipff cohort, compared to 38% in the current analysis [7, 16].

### Measuring disease activity

For the individual patient and in this prospectively followed cohort, the PedCNO score seems to be a reasonable tool for estimating ongoing changes in disease activity and treatment response. The initial findings of Beck et al. could be confirmed [10]: as shown in Table 5, the percentages of patients reaching certain levels of PedCNO score improve over time are consistent with the number of patients with decreasing activity of disease over time (as defined by MRI counted lesions; clinical lesions; PDGA). Forty percent of the patients with active disease reach a PedCNO70 during the following year. We conclude that best predictive and significantly changing parameters *for disease activity estimation during follow-up* inside the PedCNO score are MRI-defined lesions and the PGDA *over time*, in addition to the C-HAQ (Figs. 2b and 3). Even though the latter has not been designed or validated for CNO, the components of patients’ global disease estimation and pain contribute significantly to this score [17]. The C-HAQ score is known to have a ceiling effect in patients with juvenile idiopathic arthritis (JIA) and CNO [18, 19]. In our cohort, it still documents changes over time and, thus, was responsive in about half of the patients—albeit in a low score range. Patient global and pain scores alone were not responsive after 1 year of therapy and during further follow-up (Fig. 4) [20].

Pustular skin disease is a relevant component or comorbidity of CNO [4]. In the registry, the number of patients with pustulosis/acne-like skin disease did not change over time, while the proportion of IBD in CNO patients even raised during disease course.

The definition of remission in CNO is still unclear. In addition, it is unresolved, whether potential criteria for such a definition—solely or combined—would reliably describe the absence of disease activity over years. There is a definite clinical need to find a reliable and hopefully easy to use clinical severity scoring system including. We analyzed different single scores or criteria possibly describing inactive disease from both patients’ and physicians’ perspectives:

#### Patients’ pain score < 1

During 4 years of follow-up, about half of the patients reported absence of pain. As reported previously, this item changes rapidly in the first few weeks after NSAID therapy is instituted [10]. After 1 year, no further changes were noted in this low range of symptom severity. This may limit its meaningfulness and responsiveness for defining inactivity. On the other hand, patients may still feel pain after years, even though no lesions are detectable in MRI and no clinical pathologies are found. Pain amplification syndrome has been reported in CNO in this regard [21]. About half of the patients still report pain (NRS ≥ 1) after 1 year and longer at a mean NRS around 2. From the second year on, pain rating was not correlated to further improvement of numbers of lesions, neither clinically defined nor by MRI.

#### Patients’ overall well-being < 1

Almost comparable to the pain score, during the initial year this criterion covers improvement of disease but fails to describe the patients’ improvement later, especially if the PDGA would be suggested for comparison as the “gold standard of disease activity” estimation. Almost 60% of patients do not reach a patients’ global level below “one” after 4 years, while only 38% of patients had visible bone manifestations in the MRI at that time. Nevertheless, the patients’ view on her/his own disease is of utmost importance.

#### Physicians’ global disease assessment PGDA < 1

After 4 years, physicians reported “no disease activity” in 75% of patients. A PGDA < 1 may come closest to patient’s or MRI defined absent number of lesions (*n* = 0) suggesting inactive disease. Of note, certainly the PDGA is influenced by the physician’s knowledge of the MRI results as a potential bias (*r* = 0.3, Additional file 4). Most improvement of this parameter is seen in the first year of observation, and it continues to decline throughout follow-up.

#### Inactive disease as defined by absent whole body MRI lesions

Sixty percent of patients reach this target after 4 years (lesions *n* = 0). MRI definition of lesions seems more sensitive than the patients’/physicians’ clinical notice of lesions, but it may overstate the clinical relevance of a T2-positive TIRM/STIR-MRI lesion with regard to inflammation. Nevertheless, based on the literature, a T2 active lesion seems to be of relevance for the patients’ disease activity even after years of follow-up [22]. For now, it seems not entirely clear whether the mere detection of a fat saturated T2-TIRM/STIR signal necessarily implements present disease activity especially late in the CNO course [23]. Of note, 40% of the patients still show active inflammation/TIRM positive signals in MRI after 4 years, whereas 33% of patients do notice these lesions as active (Fig. 1). Thus, at least to some extent (up to 7% in the current cohort), MRI may “overstate” the activity of the lesions if the clinical notice of a lesion is prioritized.

### Predictors for severe disease course

#### Specifics of locations and number of lesions

In general, CNO affects any bone of the body [24, 25] (the neurocranium remains exceptional [26]). Therefore, a detailed analysis of the *location* of inflammation was performed. During the course of the disease, inflamed lesions were predominately present on the lower extremity and the clavicle. A risk for severe course of disease (defined by PGDA ≥ 4) was identified through statistical correlation when lesions were present in the pelvis and femur at baseline.

Patients with a higher number of lesions exhibited a prolonged and severe course of disease. Each additional affected bone at baseline increased the risk of severe disease course by 19% (OR 1.19, *p* < 0.001). A multifocal pattern (defined as lesions ≥ 2) at baseline was found to have the highest predictive value for severe disease; it increases the risk of severe disease course by 150% (OR 2.48, *p* = 0.002).

#### Laboratory parameters of inflammation

The ESR is one of five parameters in the clinical PedCNO score. Our data now shows that an elevated ESR is associated with a higher disease activity over time. Each mm/h elevation of ESR increases this risk by 3% (OR 1.03%, *p* = 0.024).

We conclude that patients with certain baseline parameters like lesions at the femur or pelvis, high number of lesions (MRI-defined), or elevated ESR have a particular higher long-term risk for severe disease. Of interest, patients with initially assumed “severe” disease due to vertebral lesions had a favorable outcome and no increased risk for a severe long-term disease. One explanation might be that these patients usually are intensively treated by using bisphosphonates. This finding underlines the importance of defining patients with a putative risk for a severe course. The current analysis suggests considering an intensified treatment for patients with multifocal lesions and femoral or pelvic lesions. Nonetheless, such considerations should be analyzed through controlled prospective trials.

### Therapy

While NSAIDs remained an important tool in treatment plans over the years, the use of steroids almost diminished completely over time in our cohort. Most patients have an uncomplicated course of disease and are treated with NSAIDs (86% initially). Generally, escalation of therapy was necessary only in a limited number of patients. These patients are often no longer treated with NSAIDs: So, in the first year, about one third of the patients is treated with DMARDs, raising up to 55% in the 4-year follow-up. In the meanwhile, 40% of patients receive NSAIDs in the fifth year. Prolonged treatment was less based on NSAIDs. However, it is still unknown whether and when stopping of NSAIDs is reasonable. The only existing prospective study by Beck et al. [10] so far pointed out NSAID effectiveness in the first year of disease [10]. Long-term data of this controlled prospective cohort is so far preliminary reported [9]. Bisphosphonate application is rather used during the first years of disease, indicating that bisphosphonates are considered predominantly for an initial “remission induction” therapy but not as a long-term continuous treatment. So, the proportion of patients with bisphosphonate treatment continuously declined, in line with fewer patients affected by lesions of the vertebrae. We consider that patients with vertebral lesions are being treated differently in the beginning compared to those patients affected by peripheral lesions. In general, patients with CNO were in a good clinical condition after 1 year (patient well-being NRS ranged around 2 (of 10) over the years; C-HAQ of zero in 2/3 of the patients after 2 years of treatment). Schnabel et al. [5] highlighted the risk of relapse in the third year of disease. To date, there is no consensus how to design an optimal disease controlling and flare preventing strategy.

When treatment strategies would only be based on patient-reported pain and numbers of clinical lesions, there may be a risk of overtreatment especially for patients affected by pain amplification syndromes or undertreatment of those with persistent bone inflammation without symptoms. On the other hand, if the presence of “active” lesions in MRI might be the only rationale of treatment, the patient may also face the risk of overtreatment in the long run. Risk factors for severe disease course (multifocal disease, inflammation of pelvis/femur at disease start, high ESR at baseline), as defined in the current study, may be considered for treatment decisions: This might lead to the assumption that intensive treatment of femur/pelvis/multifocal disease following a CNO treat-to target (T2T) strategy already in the beginning of disease might lead to a better clinical outcome for these patients, similar to the patients with vertebral lesions in this cohort. Currently, the international CARRA consensus treatment plans do not include such an additional controlled option for particular “risk” lesions, aside from the vertebral lesion [19]. It certainly seems worth defining prospective outcome parameters through larger prospective and controlled studies defining those patients, who may need particular ways of therapeutic strategies including early escalation of therapy. The NPRD cannot follow and evaluate treatment efficacy in such a controlled setting. Nevertheless, the current analysis might give implications for the set-up of treat-to-target protocols or controlled trials in international efforts [19].

### Growth development

In the previous first-year analysis of the NPRD cohort, a significant increase of CNO affected children with a growth retardation below the 3rd percentile and weight below the 3rd percentile of the standard cohort was noted, affecting around 8% of patients [2] compared to the national reference cohort [13]. The current analysis of anthropometric data (height, weight, BMI) of the cohort implies that some CNO patients start out small and may stay small and light-weighted over the time. A significant gain of weight/height after instituting effective therapy strategies, as confirmed by several means of disease activity, could not be seen in these patients. On the other hand, low height and weight at inclusion were no predictors for severe disease course. These findings allow several possible interpretations that need further investigations: some CNO patients may be affected by a constitutive energy consuming pathophysiology that is impairing appropriate growth and is still present even after effective therapy. Treatment did not alter this pattern over time. However, a further decline in length and weight was not documented. Inflammation in conjunction with metabolic energy consuming processes might play a role in CNO [27, 28]. Due to the limited number of patients, this finding may not be overinterpreted. So far, only a few patients with a monogenetic disease background mimicking CNO and also affecting growth restrictions have been reported, like hypophosphatasia and Majeed syndrome [28, 29]. In daily pediatric practice, low weight and height should prompt the caring physician to consider further metabolic or genetic diagnostic approaches in CNO. In addition, these findings emphasize the necessity of an early CNO diagnosis and treatment to prevent a possible further decline in growth characteristics.

### Limitations of the analysis

In comparison to other cohorts, the patients in the registry do have a slightly lower average MRI lesion number. Possible causes are the delay of inclusion into the registry (5.8 months after first contact to pediatric rheumatologist). There still might be a bias by the shrinking size of patient numbers/lesions over time by losing manifestation locations with lower frequencies. There was a remarkable number of patients who was not followed for the entire 4 years in the registry. We could not find any sociodemographic or clinical parameter that was associated with the likelihood of drop-out. Therefore, the longitudinal data analysis by linear mixed models results in unbiased effect estimates in presence of missing parameters. In addition, we cannot provide data about treatment effectiveness and reasons for discontinuation in this longitudinal data analysis. Measurements of disease activity at treatment start as well as reasons for discontinuation are not collected; only the presentation of patients at the visit is documented in the NPRD.

## Conclusion

A subgroup of CNO patients still remains in pediatric rheumatologic care for years with the need of medication, while a majority of CNO patients experiences inactive disease with no need of therapeutic intervention over time. We were able to identify outcome predictors for severe disease at disease onset like multifocal disease, elevated ESR, and certain bone lesions. Future investigations may help confirming these predictors in order to justify early treatment decisions. PedCNO score and especially PGDA, MRI-defined lesions, and in a number of patients also the C-HAQ appear to be promising parameters for describing disease activity. These findings may be important for patients at risk for severe and prolonged disease and influence treatment decisions.

### Supplementary Information

**Additional file 1.** Development of physician global, pain, overall well-being (NRS 0-10), ESR and C-HAQ (0-3) from inclusion and 4 years of follow-up. NRS: numeric rating scale; ESR: erythrocyte sedimentation rate, C-HAQ: childhood Health assessment questionnaire; YFU: year follow-up. Inclusion 5.8 months after first visit to pediatric rheumatology. The values are given as mean and, in brackets, standard deviation.

**Additional file 2.** Scores suggesting inactive disease. Physician global disease activity (PGDA) NRS <1, patient pain NRS <1, patient overall well-being NRS <1 and C-HAQ =0 (childhood health assessment questionnaire) from inclusion to 4 years of follow-up; percentages of patients are given, who reached the proposed levels of remission. OR odds ratio. NRS: numeric rating scale

**Additional file 3.** Comparison of different cohorts. Expanded and adapted from [2].

**Additional file 4.** Correlation of disease activity measures with each other over 4 years follow-up. C-HAQ: childhood Health assessment questionnaire. MRI: magnetic resonance imaging. ESR: erythrocyte sedimentation rate. PGDA: physician global disease activity

**Additional file 5.** List of participating centers.

## Data Availability

The datasets used and/or analyzed during the current study are available from the corresponding author on reasonable request.

## Abbreviations

BMI: Body mass index
bDMARD: Biological disease-modifying anti-rheumatic drug
CARRA: Childhood Arthritis and Rheumatology Research Alliance
C-HAQ: Childhood Health Assessment Questionnaire
CNO: Chronic nonbacterial osteomyelitis
CRMO: Chronic recurrent multifocal osteomyelitis
CRP: C-reactive protein
csDMARD: Conventional disease-modifying anti-rheumatic drug
CTPs: Consensus treatment plans
DMARDs: Disease-modifying anti-rheumatic drugs
ESR: Erythrocyte sedimentation rate
ERA: Enthesitis-related arthritis
HLA: Human leukocyte antigen
ILAR: International league against rheumatism
MRI: Magnetic resonance imaging
MTX: Methotrexate
NPRD: National Pediatric Rheumatologic Database
NRS: Numeric rating scale
NSAIDs: Nonsteroidal anti-inflammatory drugs
PedCNO: Pediatric chronic nonbacterial osteomyelitis follow-up treatment score
PGDA: Physician global disease activity
QoL: Quality of life
SAPHO: Synovitis acne pustulosis hyperostosis osteitis syndrome
SD: Standard deviation
SPA: Spondyloarthropathy
STD: Standard deviation
STIR: Short tau inversion recovery
T2T: Treat-to- target recommendations
TIRM: Turbo inversion recovery measurement
TNF: Tumor necrosis factor
VAS: Visual analogue scale
WB-MRI: Whole-body magnetic resonance imaging
YFU: Years follow-up
