# Supplementary material for: Long-term follow-up of children with chronic non-bacterial osteomyelitis—assessment of disease activity, risk factors, and outcome
Source: Arthritis Res Ther. 2023 Nov 28;25:228. doi: 10.1186/s13075-023-03195-4 (PMC10683360; doi:10.1186/s13075-023-03195-4)
Supplement: Supplementary file 1 — Additional file 1. Development of physician global, pain, overall well-being (NRS 0-10), ESR and C-HAQ (0-3) from inclusion and 4 years of follow-up. NRS: numeric rating scale; ESR: erythrocyte sedimentation rate, C-HAQ: childhood Health assessment questionnaire; YFU: year follow-up. Inclusion 5.8 months after first visit to pediatric rheumatology. The values are given as mean and, in brackets, standard deviation. [file 13075_2023_3195_MOESM1_ESM.docx]

|  | Inclusion | 1 YFU | 2 YFU | 3 YFU | 4 YFU | beta | 95% CI | p-value |
| --- | --- | --- | --- | --- | --- | --- | --- | --- |
| physician global disease activity (NRS) | 2.1 (1.9) | 1.2 (1.5) | 1.0 (1.5) | 1.0 (1.4) | 0.9 (1.7) | -0.13 | -0.32 ; -0.06 | 0.018 |
| ESR in mm/h | 17.5 (16.0) | 12.9 (12.7) | 12.1 (12.1) | 11.0 (7.9) | 13.1 (12.3) | -2.07 | -2.88 ; -1.28 | <0.001 |
| patient pain (NRS) | 2.7 (2.8) | 2.0 (2.5) | 1.9 (1.5) | 2.1 (2.6) | 1.9 (2.6) | -0.16 | -0.36 ; 0.05 | 0.134 |
| patient overall well-being (NRS) | 2.7 (2.5) | 1.9 (2.1) | 2.0 (2.2) | 2.1 (2.4) | 2.0 (2.5) | -0.71 | -1.23 ; -0.20 | 0.007 |
| C-HAQ | 0.3 (0.4) | 0.2 (0.3) | 0.1 (0.3) | 0.2 (0.4) | 0.1 (0.3) | -0.04 | -0.06 ; -0.02 | <0.001 |
