# Supplementary material for: Long-term follow-up of children with chronic non-bacterial osteomyelitis—assessment of disease activity, risk factors, and outcome
Source: Arthritis Res Ther. 2023 Nov 28;25:228. doi: 10.1186/s13075-023-03195-4 (PMC10683360; doi:10.1186/s13075-023-03195-4)
Supplement: Supplementary file 2 — Additional file 2. Scores suggesting inactive disease. Physician global disease activity (PGDA) NRS <1, patient pain NRS <1, patient overall well-being NRS <1 and C-HAQ =0 (childhood health assessment questionnaire) from inclusion to 4 years of follow-up; percentages of patients are given, who reached the proposed levels of remission. OR odds ratio. NRS: numeric rating scale [file 13075_2023_3195_MOESM2_ESM.docx]

|  | **Inclusion/**  **5.8 months after first visit** | **1YFU** | **2YFU** | **3YFU** | **4YFU** | **OR^1^** | **95%CI** | **p- value** |
| --- | --- | --- | --- | --- | --- | --- | --- | --- |
| **PGDA< 1** | 130  (33.9%) | 148  (50.0%) | 135 (57.7%) | 78 (57.8%) | 54 (72%) | **1.39** | **1.27 ; 1.52** | **<.0001** |
| **Patients’ pain < 1** | 130  (36.2%) | 130 (48.9%) | 77 (47.4%) | 59 (50.9%) | 39 (52.0%) | **1.19** | **1.08 ; 1.31** | **0.005** |
| **Patients’ overall well being < 1** | 98  (27.5%) | 103 (39.2%) | 77 (36.5%) | 39 (33.3%) | 29 (38.7%) | **1.11** | **1.02 ; 1.21** | **0.017** |
| **C-HAQ = 0** | 170  (47.1%) | 163 (60.6%) | 145 (68.4%) | 78 (65.0%) | 52 (70.3%) | **1.33** | **1.20 ; 1.48** | **<.0001** |
