# Supplementary material for: Long-term follow-up of children with chronic non-bacterial osteomyelitis—assessment of disease activity, risk factors, and outcome
Source: Arthritis Res Ther. 2023 Nov 28;25:228. doi: 10.1186/s13075-023-03195-4 (PMC10683360; doi:10.1186/s13075-023-03195-4)
Supplement: Supplementary file 3 — Additional file 3. Comparison of different cohorts. Expanded and adapted from [2]. [file 13075_2023_3195_MOESM3_ESM.docx]

|  | Canada 2002 (16) | USA  2012 (6) | France 2015 (7) | UK  2016 (8) | Germany 2015 (9) | Eurofever 2017 (4) | German NPRD 2020 (2) | German NPRD 2023 |
| --- | --- | --- | --- | --- | --- | --- | --- | --- |
| Number of Patients | 23 | 70 | 178 | 41 | 95 | 486 | **774** | **400** |
| male % | 17 | 33 | 31 | 25 | 42 | 36 | **37** | **36** |
| female % | 83 | 67 | 69 | 75 | 58 | 64 | **63** | **64** |
| mean age at disease onset | 9 | 9.7 | 10.9 | 9 | 11.7 | 9.9 | **11.1** | **11.1** |
| delay of diagnosis in months | 13 | 6 | 17 | 15 | 11 | 12 | **8.4** | **5** |
| number of lesions, mean | 4 | 3.5 | 3 | N/A | 4 | 4 | **3** | **3 (MRI)**  **2 (clinical)** |
| unifocal in % | 43 | 29 | 7 | 24 | 16 | 29 | **37** | **35 (MRI)** |
| multifocal in % | 57 | 71 | 93 | 76 | 84 | 71 | **63** | **65 (MRI)** |
| active disease in % after follow up | 22 | 39 | 66 | N/A | 33 | 50 | **47** | **38** |
| months of follow up | 68 | 22 | 48 | N/A | 49 | N/A | **12** | **48** |
