# Supplementary material for: Long-term follow-up of children with chronic non-bacterial osteomyelitis—assessment of disease activity, risk factors, and outcome
Source: Arthritis Res Ther. 2023 Nov 28;25:228. doi: 10.1186/s13075-023-03195-4 (PMC10683360; doi:10.1186/s13075-023-03195-4)
Supplement: Supplementary file 4 — Additional file 4. Correlation of disease activity measures with each other over 4 years follow-up. C-HAQ: childhood Health assessment questionnaire. MRI: magnetic resonance imaging. ESR: erythrocyte sedimentation rate. PGDA: physician global disease activity [file 13075_2023_3195_MOESM4_ESM.docx]

| **Correlations** | **PGDA** | **patient's overall well-being** | **Patient’s pain** | **C-HAQ** | **# clinical lesions** | **# MRI lesions** | **ESR** |
| --- | --- | --- | --- | --- | --- | --- | --- |
| **PGDA** | 1.00 | 0.40 | 0.43 | 0.25 | 0.34 | 0.30 | 0.29 |
| **patient's overall well-being** |  | 1.00 | **0.77** | **0.49** | 0.18 | 0.10 | 0.19 |
| **Patient’s pain** |  |  | 1.00 | 0.48 | 0.17 | 0.07 | 0.13 |
| **C-HAQ** |  |  |  | 1.00 | 0.10 | 0.06 | 0.11 |
| **# clinical lesions** |  |  |  |  | 1.00 | 0.42 | 0.17 |
| **# MRI lesions** |  |  |  |  |  | 1.00 | 0.09 |
| **ESR** |  |  |  |  |  |  | 1.00 |
