# Supplementary material for: Long-term follow-up of children with chronic non-bacterial osteomyelitis—assessment of disease activity, risk factors, and outcome
Source: Arthritis Res Ther. 2023 Nov 28;25:228. doi: 10.1186/s13075-023-03195-4 (PMC10683360; doi:10.1186/s13075-023-03195-4)
Supplement: Supplementary file 5 — Additional file 5. List of participating centers. [file 13075_2023_3195_MOESM5_ESM.docx]

Tomas Berger, Vestische Kinder- und Jugendklinik Datteln, Rheumatologie/Immunologie, Datteln; Rainer Berendes, Kinderklinik St. Marien, Landshut; Regine Borchers, Universitätsklinikum Augsburg, Klinik für Kinder- und Jugendliche, Augsburg; Michael Borte, Städtisches Klinikum St. Georg, Klinik für Kinder- und Jugendmedizin, Leipzig; Jürgen Brunner, Medizinische Universität Innsbruck, Kinder- und Jugendheilkunde, Innsbruck; Frank Dressler, Medizinische Hochschule Hannover, Kinderklinik, Hannover; Ivan Foeldvari, Hamburger Zentrum für Kinder- und Jugendrheumatologie, Schwerpunktpraxis am Klinikum Eilbek, Hamburg; Dirk Föll, Universitätsklinik Münster, Klinik für Pädiatrische Rheumatologie und Immunologie, Münster; Anja Fröhlich, Universitätsklinik Eppendorf, Klinik und Poliklinik für Kinder- und Jugendmedizin, Hamburg; Matthias Galiano, Universitätsklinikum Erlangen, Kinder- und Jugendklinik, Erlangen; Hermann Girschick, Vivantes Klinikum Friedrichshain, Berlin; Jürgen Grulich-Henn, Universitätsklinikum Heidelberg, Zentrum für Kinderund Jugendmedizin - Kinderheilkunde I, Heidelberg; Johannes-Peter Haas, Deutsches Zentrum für Kinderund Jugendrheumatologie, Garmisch-Partenkirchen; Maria Haller, Kinderarztpraxis, Gundelfngen; Georg Heubner, Städtisches Klinikum Dresden-Neustadt, Klinik für Kinder- und Jugendmedizin, Dresden; Nadja Hofmann, Sozialstiftung Bamberg, Klinik für Kinder und Jugendliche, Bamberg; Anette Holl-Wieden, Universitätsklinikum Würzburg, Kinderklinik und Poliklinik, Würzburg; Gerd Hornef, Asklepios Kinderklinik St. Augustin, Zentrum für Allgemeine Pädiatrie und Neonatologie, Sankt Augustin; Anton Hospach, Zentrum für Pädiatrie, Olgahospital, Klinikum Stuttgart; Regina Hühn, Martin-Luther-Universität Halle-Wittenberg, Halle (Saale); Markus Hufnagel, Zentrum für Kinder- und Jugendmedizin, Universitätsklinikum, Freiburg; Ales Janda, Universitätsklinikum Ulm, Klinik für Kinder- und Jugendmedizin, Ulm; Annette Jansson, Dr.-von-Haunersches Kinderspital der LMU, Kinderklinik und Kinderpoliklinik, München; Tilmann Kallinich, Universitätsmedizin Berlin - Charité, Campus Virchow-Klinikum, Otto-Heubner-Centrum für Kinder- und Jugendmedizin, Berlin; Tomas Keller, Josefnum Krankenhaus, Klinik für Kinder und Jugendliche, Augsburg; Hans Kössel, Klinikum Westbrandenburg, Kinder- und Jugendmedizin, Brandenburg; Elke Lainka, Universitäts-Kinderklinik Essen, Zentrum für Kinderund Jugendmedizin, Essen; Georg Leipold, Gemeinschaftspraxis Kinder- und Jugendärzte, Regensburg; Jan Maier, Kinderarztpraxis, Leinfelden-Echterdingen; Kristina Mathony, Städtisches Klinikum Dessau, Klinik für Kinder- und Jugendmedizin, Dessau; Almut MeyerBahlburg, Universitätsmedizin Greifswald, KöR, Abt. Allgemeine Pädiatrie, Greifswald; Kirsten Minden, Universitätsmedizin Berlin - Charité, Campus VirchowKlinikum, Otto-Heubner-Centrum für Kinder- und Jugendmedizin, Berlin; Kirsten Mönkemöller, Kinderkrankenhaus der Stadt Köln, Kinder- und Jugendmedizin, Köln; Tim Niehues, Helios Klinikum Krefeld, Pädiatrische Institutsambulanz, Krefeld; Nils Onken, Kinderarztpraxis, Lüneburg; Prassad Oommen, Med. Einrichtungen der Heinrich-Heine-Universität, Zentrum für Kinder -und Jugendmedizin, Düsseldorf; Claudia Präger, Diakonie-Klinikum Schwäbisch Hall, Kinderklinik, Schwäbisch Hall; Jürgen Quietzsch, DRK Krankenhaus Lichtenstein, Klinik für Kinder- und Jugendmedizin, Lichtenstein; Christiane Reiser, Kinderklinik, Landeskrankenhaus Bregenz, Bregenz; Christoph Rietschel, Clementine Kinderhospital, Klinik für Kinder- und Jugendmedizin, Frankfurt; Betina Rogalski, Kinderrheumatologische Privatpraxis, Alsbach-Hähnlein; Michael Rühlmann, Kinderarztpraxis, Göttingen; Peggy Rühmer, Helios Vogtland-Klinikum Plauen, Fachambulanz der Klinik für Kinder- und Jugendmedizin, Plauen; Axel Sauerbrey, Helios Klinikum Erfurt, Klinik für Kinder- und Jugendmedizin, Erfurt; Anja Schnabel, Universitätsklinikum Carl Gustav Carus, Klinik und Poliklinik für Kinder- und Jugendmedizin, Dresden; Martin Scholten, Universitätsklinikum Jena, Klinik für Kinder- und Jugendmedizin, Jena; Volker Schuster, Universitätsklinik und Poliklinik für Kinder und Jugendliche, Rheumaambulanz, Leipzig; Catharina Schütz, Universitätsklinikum Carl Gustav Carus, Klinik und Poliklinik für Kinder- und Jugendmedizin, Dresden; Anja Sonnenschein, JohannGutenberg-Universität Mainz, Zentrum für Kinder- und Jugendmedizin, Mainz; Claudia Stollbrink, Universitätsklinikum Aachen; Kinderklinik RWTH Aachen, Klinik für Kinder- und Jugendmedizin, Aachen; Ralf Trauzeddel, Helios Klinikum Berlin-Buch, Klinik für Kinderund Jugendmedizin, Berlin; Philipp von Bismarck, Universitätsklinikum Schleswig Holstein - Campus Kiel, Klinik für Kinder- und Jugendmedizin, Kiel; Frank Weller-Heinemann, Klinikum Bremen Mitte - ProfessorHess-Kinderklinik, Zentrum für Kinder- und Jugendrheumatologie, Bremen; Daniel Windschall, St. Josef-Stift Sendenhorst, Abt. Kinder- und Jugendrheumatologie, Sendenhorst
